# Supplementary material for: Type-2 CD8+ T-cell formation relies on interleukin-33 and is linked to asthma exacerbations
Source: Nat Commun. 2023 Aug 23;14:5137. doi: 10.1038/s41467-023-40820-x (PMC10447424; doi:10.1038/s41467-023-40820-x)
Supplement: Supplementary file 1 — Supplementary Information [file 41467_2023_40820_MOESM1_ESM.pdf]

Type-2 CD8+ T cell formation relies on interleukin-33 and is linked to asthma exacerbations

Supplementary Information File

AUTHORS

Esmee K. van der Ploeg[1,2], Lisette Krabbendam[1$], Heleen Vroman[1$], Menno van Nimwegen[1], Marjolein J.W. de Bruijn[1], Geertje M. de Boer[1,3], Ingrid M. Bergen[1], Mirjam Kool[1], Gerdien A. Tramper-Standers[4,5], Gert-Jan Braunstahl[1,3], Danny Huylebroeck[2], Rudi W. Hendriks[1*], Ralph Stadhouders[1,2*]

AFFILIATIONS

[1]Department of Pulmonary Medicine, Erasmus MC, University Medical Center, Rotterdam, The Netherlands

[2]Department of Cell Biology, Erasmus MC, University Medical Center, Rotterdam, The Netherlands

[3]Department of Respiratory Medicine, Franciscus Gasthuis and Vlietland, Rotterdam, The Netherlands

[4]Department of Pediatric Medicine, Franciscus Gasthuis and Vlietland, Rotterdam, The Netherlands

[5]Department of Neonatology, Sophia Children's Hospital, Erasmus MC, University Medical Center, Rotterdam, The Netherlands

[$]These authors contributed equally

*These authors jointly supervised this work

Corresponding author: r.stadhouders@erasmusmc.nl

CONTENTS

Supplementary Figures 1-11

Supplementary Tables 1-5

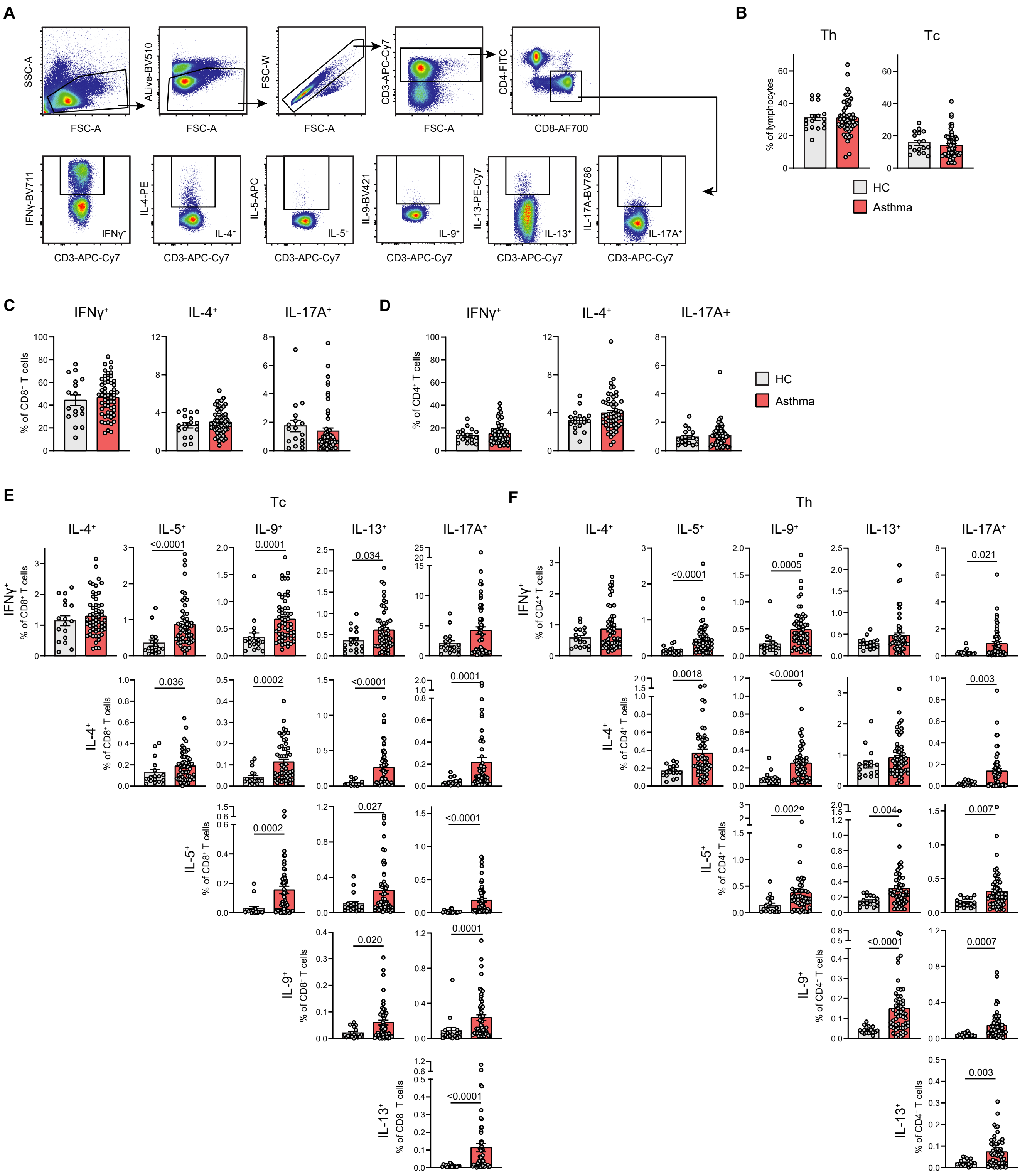

Figure S1. Type-2 skewing of circulating Tc cells in asthma. (A) Flow cytometry gating strategy used to identify IFNγ⁺, IL-4⁺, IL-5⁺, IL-9⁺, IL-13⁺ and IL-17A⁺ CD4⁺ and CD8⁺ T cells (gating for Tc cells is depicted). This gating strategy is applied for the following figures: 1A-D, 2A-F, S1B-F, S2A-D, S3A-B, S4A-C, S5A-I. **(B)** Quantification of Th and Tc cells as percentage of all lymphocytes using flow cytometry in PB samples of 17 HC individuals and 55 asthma patients. **(C-D)** Quantification of IFNγ⁺, IL-4⁺, and IL-17A⁺ Tc (panel C) and Th cells (panel D) using flow cytometry in PB samples of 17 HC individuals and 55 asthma patients. **(E-F)** Quantification of all double-producing cytokine combinations by Tc cells (panel E) and Th cells (panel F) in PB samples of 17 HC individuals and 55 asthma patients. Symbols represent individual donors; bars indicate mean values +/- SEM. Mann-Whitney U test two-tailed. PB, peripheral blood; HC, healthy control. Source data are provided as a Source Data file

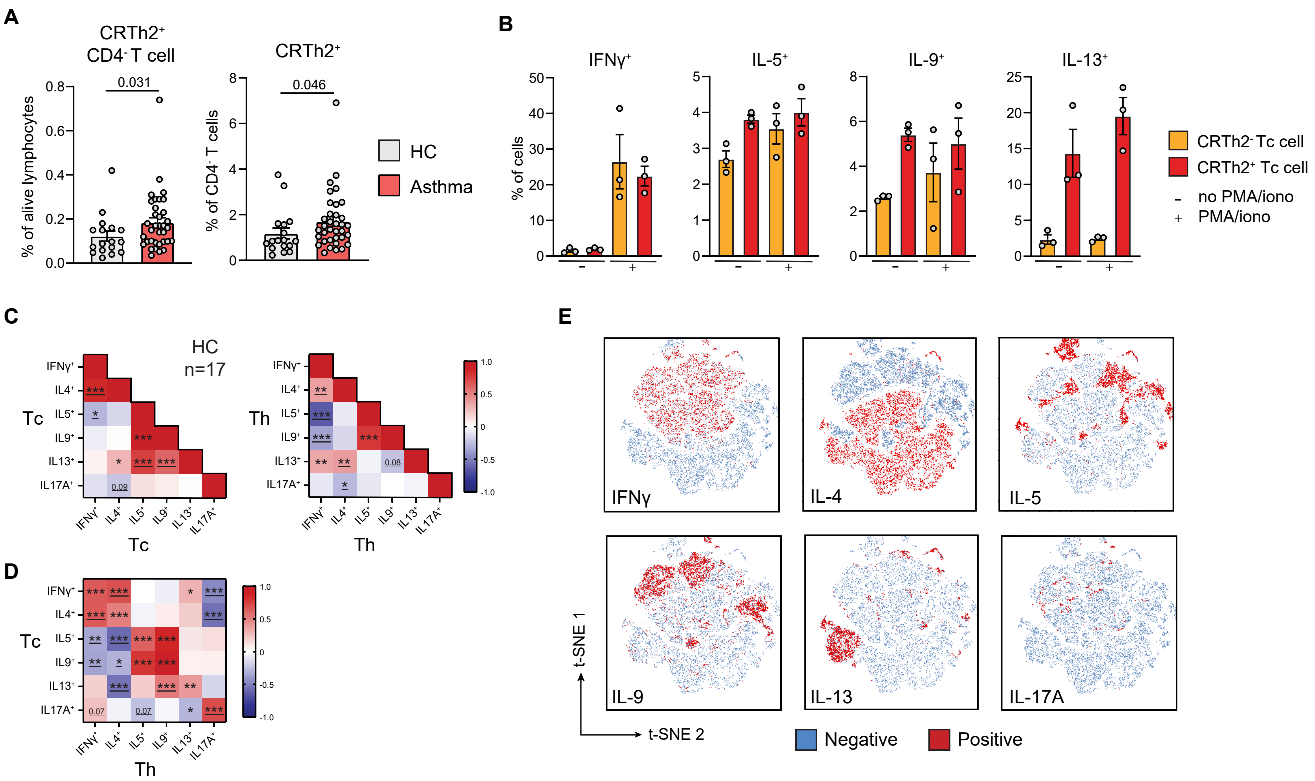

Figure S2. Type-2 cytokine production by CD8 T cells. (A) Quantification of CRTH2$^+$ CD4$^-$ T cells in PB samples of 17 HC and 35 asthma patients as percentages of lymphocytes and as percentages of CD4$^-$ T cells. **(B)** IFNγ$^+$, IL-5$^+$, IL-9$^+$ and IL-13$^+$ production by CRTH2$^-$ and CRTH2$^+$ Tc cells with and without PMA/ionomycin stimulation using flow cytometry. n=3 individuals. **(C-D)** Correlation matrices of cytokine production between (panel C) and across (panel D) Th/Tc cell compartments of 17 HCs. Underlined asterisks: difference in significance compared to asthmatics (Fig.1C-D). **(E)** tSNE analysis of flow cytometry data using all cytokine producing Th cells from asthma patients, depicting either IFNγ$^+$, IL-4$^+$, IL-5$^+$, IL-9$^+$, IL-13$^+$ or IL-17A$^+$ producing Th cells in red. Symbols in panels A-B represent individual donors; bars indicate mean values +/- SEM. *P<0.05, **P<0.01, ***P<0.001, ****P<0.0001 (Mann-Whitney U test two-tailed, Pearson correlation coefficient). PB, peripheral blood; HC, healthy control. Source data are provided as a Source Data file

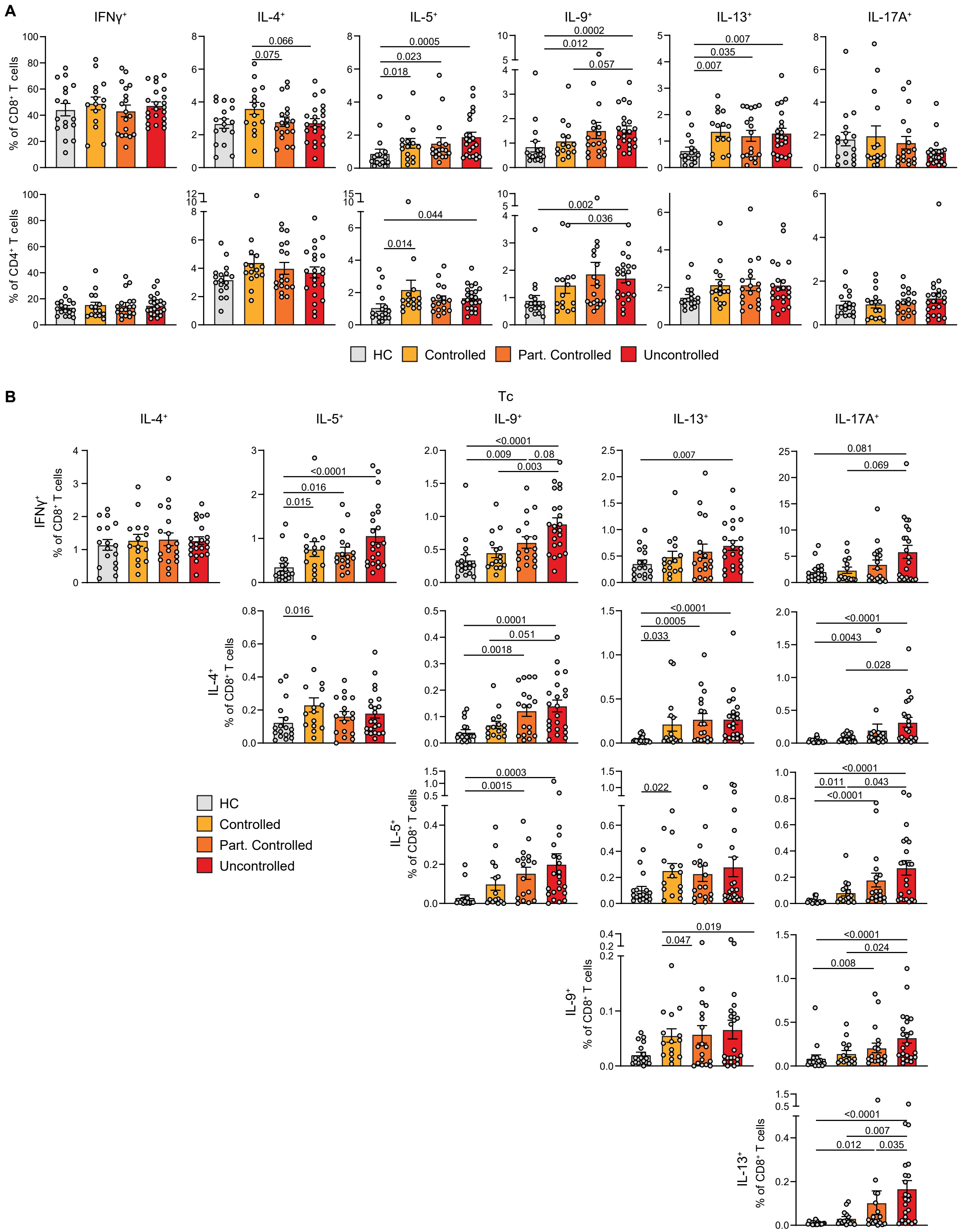

Figure S3. Type-2 skewing of Tc cells is linked to uncontrolled asthma. (A) Quantification of IFNγ+, IL-4+, IL-5+, IL-9+, IL-13+ and IL-17A+ Tc and Th cells using flow cytometry in PB samples of 17 HC individuals, 15 controlled asthma (ACQ<0.75), 18 partially controlled asthma (ACQ 0.75-1.5), and 22 uncontrolled asthma (ACQ>1.5) patients. **(B)** Quantification of all double-producing cytokine combinations by Tc cells in PB samples of 17 HC individuals, 15 controlled asthma, 18 partially controlled asthma, and 22 uncontrolled asthma patients. Symbols represent individual donors; bars indicate mean values +/- SEM. *P<0.05, **P<0.01, ***P<0.001, ****P<0.0001 (Kruskal-Wallis test corrected for multiple testing). PB, peripheral blood; HC, healthy control; ACQ, asthma control questionnaire. Source data are provided as a Source Data file

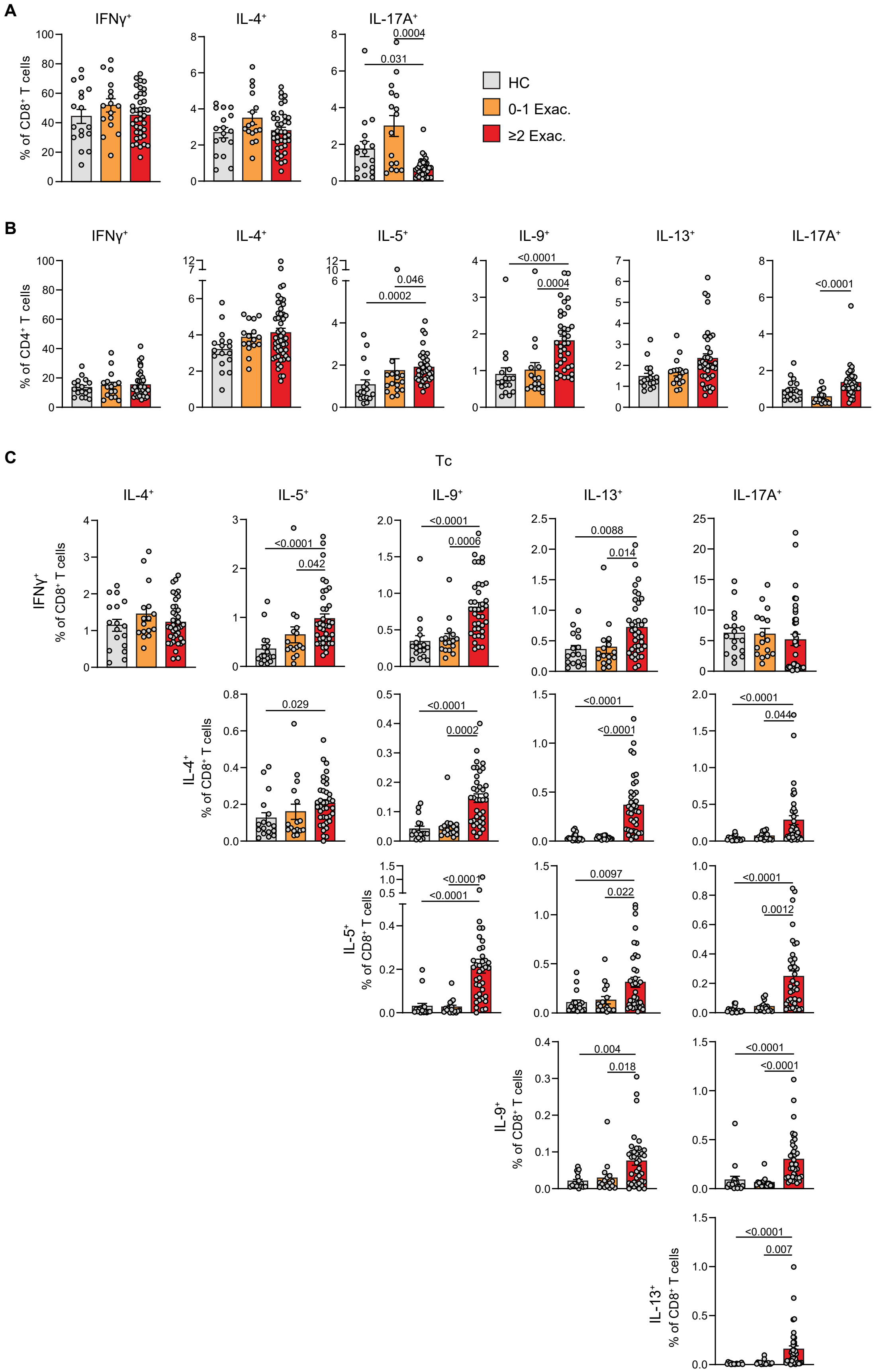

Figure S4. Type-2 skewing of Tc cells is linked to asthma exacerbations. (A) Quantification of IFNγ+, IL-4+, and IL-17A+ Tc cells using flow cytometry in PB samples of 17 HC individuals and 16 asthma patients with 0-1 exacerbations ('Exac.') or 38 patients with ≥2 exacerbations in the previous year. **(B)** Quantification of IFNγ+, IL-4+, IL-5+, IL-9+, IL-13+, and IL-17A+ Th cells using flow cytometry in PB samples of HC individuals and 16 asthma patients with 0-1 exacerbations ('Exac.') or 38 patients with ≥2 exacerbations in the previous year. **(C)** Quantification of all double-producing cytokine combinations by Tc cells in PB samples of 17 HC individuals and 16 asthma patients with 0-1 exacerbations ('Exac.') or 38 patients with ≥2 exacerbations in the previous year. Symbols represent individual donors; bars indicate mean values +/- SEM. Kruskal-Wallis test corrected for multiple testing. PB, peripheral blood; HC, healthy control. Source data are provided as a Source Data file

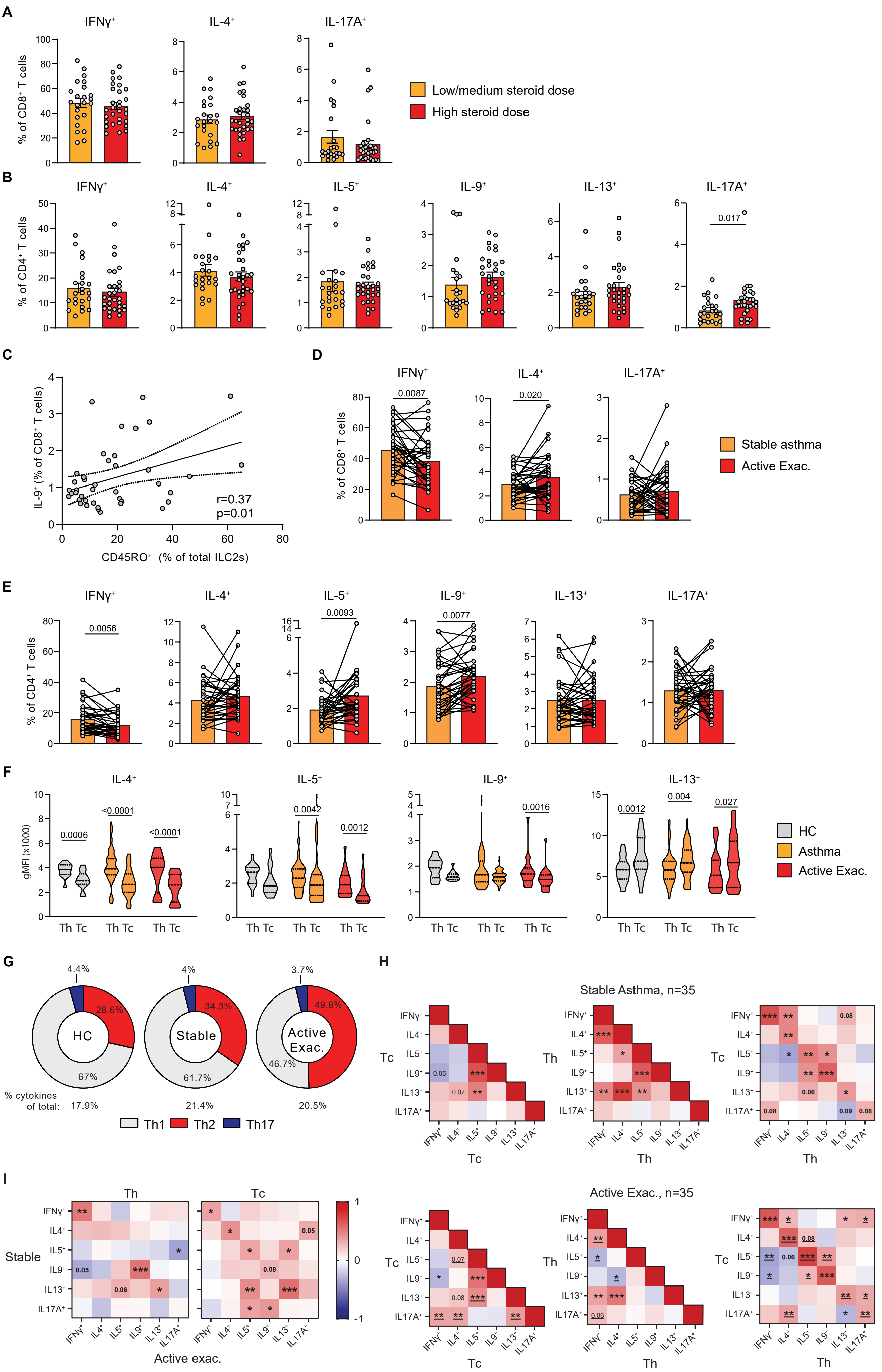

Figure S5. Type-2 skewing of Tc cells is linked to high inhaled corticosteroid dose. (A) Quantification of IFNγ$^+$, IL-4$^+$, and IL-17A$^+$ Tc cells using flow cytometry in PB samples of 23 asthma patients with low/medium steroid dose intake (<1000 bioequivalent units) and 30 patients with high steroid dose intake (>1000 bioequivalent units). **(B)** Quantification of IFNγ$^+$, IL-4$^+$, IL-5$^+$, IL-9$^+$, IL-13$^+$, and IL-17A$^+$ Th cells using flow cytometry in PB samples of 23 asthma patients with low/medium steroid dose intake (<1000 bioequivalent units) and 30 patients with high steroid dose intake (>1000 bioequivalent units). **(C)** Correlation between circulating IL-9$^+$ Tc cell frequencies and CD45RO$^+$ ILC2s frequencies across the asthma patient cohort (n=41). **(D)** Quantification of IFNγ$^+$, IL-4$^+$, and IL-17A$^+$ Tc cells in 35 paired PB samples of asthma patients during stable disease and during an active exacerbation ('Active Exac'). **(E)** Quantification of IFNγ$^+$, IL-4$^+$, IL-5$^+$, IL-9$^+$, IL-13$^+$, and IL-17A$^+$ Th cells using flow cytometry in 35 paired PB samples of asthma patients during stable disease and during an active exacerbation ('Active Exac'). **(F)** geometric Mean Fluorescent Intensity (gMFI) of IL-4$^+$, IL-5$^+$, IL-9$^+$, and IL-13$^+$ Tc and Th cells in 17 HC, 67 asthma patients and in asthma patients during an active exacerbation ('Active Exac', n=20). **(G)** Pie charts summarizing proportions of Th1, Th2 and Th17 cells in HCs, asthma patients, and during an active exacerbation. '% cytokines of total:' indicates percentage all Tc cells that produce one of the cytokines assayed. **(H)** Correlation matrices of cytokine production between Th and Tc cells in stable asthma or during active exacerbations (n=35). **(I)** Correlation matrices of cytokine production by Th (left) and Tc (right) cells across stable asthma and active exacerbations (n=35). Symbols in panels A-E represent individual donors; bars indicate mean values +/- SEM. *P<0.05, **P<0.01, ***P<0.001, ****P<0.0001 (Mann-Whitney U test two-tailed, Wilcoxon-paired test two-tailed or Wilcoxon rank-sum test two-tailed), underlined asterisks in panels H and I: difference in significance compared to stable asthmatics. PB, peripheral blood; HC, healthy control; Th1: cells producing IFNγ excluding double-producers of type-2 or type-3 cytokines; Th2: any cell producing either IL-4, IL-5, IL-9, or IL-13; Th17: cells producing IL-17A excluding double-producers of type-1 or type-2 cytokines. PB, peripheral blood; HC, healthy control. Source data are provided as a Source Data file

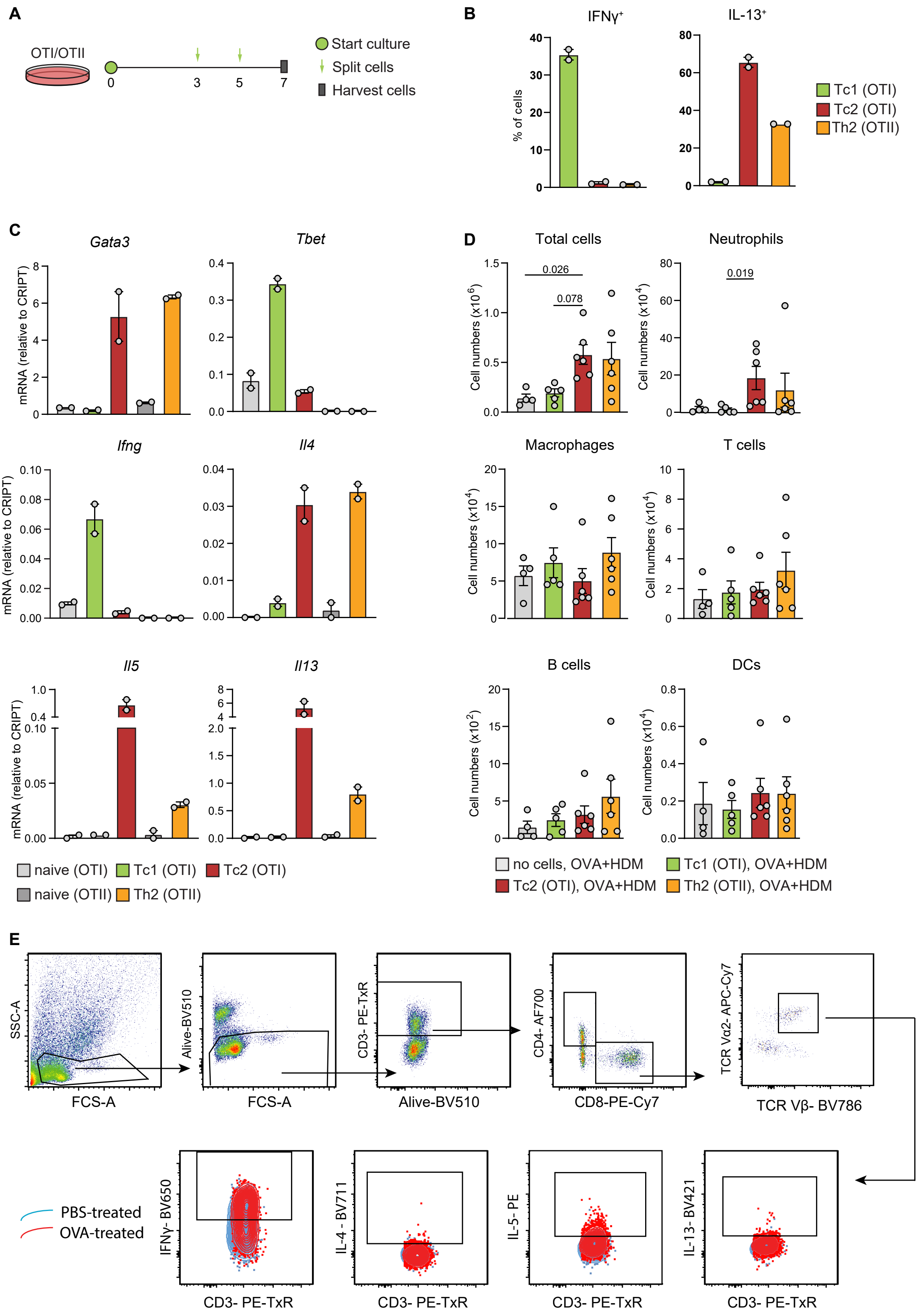

Figure S6. Eosinophilic airway inflammation induced by transferred Tc2 cells. (A) Schematic overview of in vitro culture protocol of splenic OTI/OTII T cells to generate Tc1, Tc2 and Th2 cells. Isolated cells were sorted and cultured with anti-CD3, anti-CD28 for 7 days in the presence of IL-2 for Tc1 polarization, or IL-2, IL-4 and anti–IFNγ for Tc2 polarization. For Th2 polarization, cells were cultured in the presence of IL-2, IL-4, anti–IFNγ and anti-IL-12/23 p40. **(B)** Flow cytometry analysis of IFNγ and IL-13 production by cultured OTI/OTII cells. **(C)** Quantitative PCR analysis of *Gata3, Tbet, Ifng, Il4, Il5,* and *Il13* present in cultured OTI/OTII cells. **(D)** Numbers of total cells, neutrophils, macrophages, T cells, B cells, and total DCs determined in BAL by flow cytometry analysis. no cells_OVA+HDM: n=4, OTI Tc1_OVA+HDM: n=5, OTI Tc2_OVA+HDM: n=6, OTII Th2_OVA+HDM: n= 6. **(E)** Flow cytometry gating strategy used to identify cytokine production by Th and Tc cells in Figure 3D. Symbols represent individual mice; bars indicate mean values +/- SEM. *P<0.05 (Kruskal-Wallis test corrected for multiple testing). OVA, Ovalbumine. Source data are provided as a Source Data file

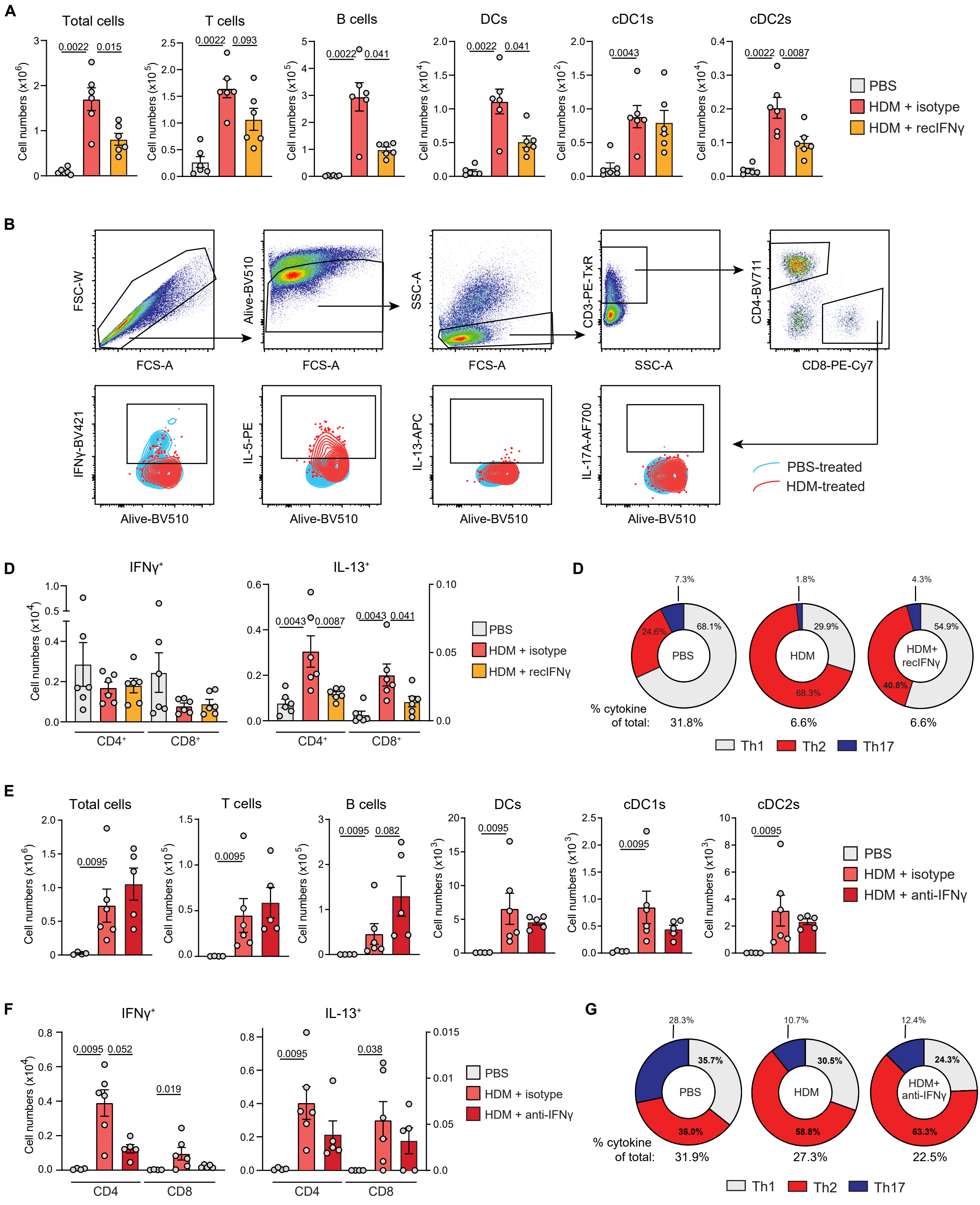

Figure S7. IFNγ signaling suppresses type-2 skewing of Tc cells. (A) Numbers of total cells, T cells, B cells, total DCs, cDC1s, and cDC2s determined in BAL by flow cytometry analysis. n=6 mice per group **(B)** Flow cytometry gating strategy used to identify cytokine production by Th and Tc cells in Figure 4C-E and S7D. **(C)** Numbers of IFNγ^{+} and IL-13^{+} Th and Tc cells in BAL measured by flow cytometry. B-D: n=6 mice per group **(D)** Pie charts summarizing proportions of Th1, Th2 and Th17 cells in PBS or HDM-sensitized WT mice treated with or without recIFNγ. **(E)** Numbers of total cells, T cells, B cells, total DCs, cDC1s, and cDC2s determined in BAL by flow cytometric analysis. **(F)** Numbers of IFNγ^{+} and IL-13^{+} Th and Tc cells in BAL by flow cytometry. **(G)** Pie charts summarizing proportions of Th1, Th2 and Th17 cells in PBS or HDM-sensitized WT mice treated with or without anti-IFNγ. '% cytokine of total:' indicates percentage all Th cells that produce one of the cytokines assayed. Symbols represent individual mice, E-F: PBS: n=4, HDM: n=6, HDM+anti-IFNγ: n= 5, bars indicate mean values +/- SEM. Mann-Whitney U test two-tailed. AAI, allergic airway inflammation; HDM, house dust mite; WT, wild type; BAL: bronchoalveolar lavage. Source data are provided as a Source Data file

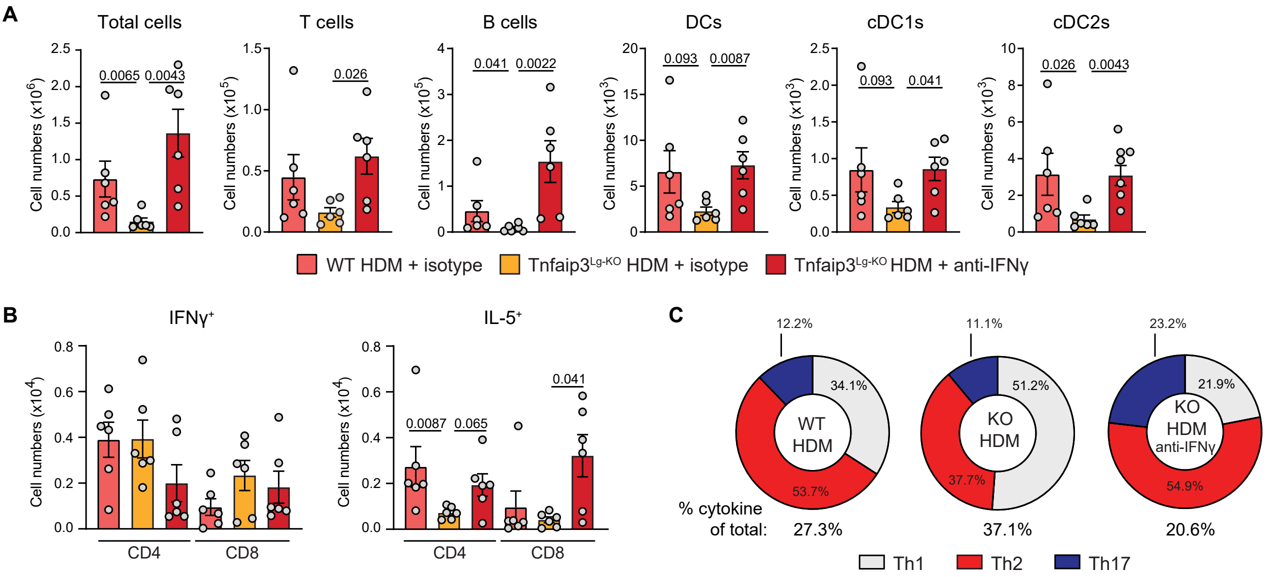

Figure S8. Depletion of *Tnfaip3* in Langerin⁺ cDC1s suppresses type-2 skewing of Tc cells which is restored by blocking IFNγ. (A) Numbers of total cells, T cells, B cells, total DCs, cDC1s, and cDC2s determined in BAL by flow cytometry analysis. **(B)** Numbers of IFNγ⁺ and IL-5⁺ Th and Tc cells in BAL measured by flow cytometry. **(C)** Pie charts summarizing proportions of Th1, Th2 and Th17 cells in HDM-sensitized WT or *Tnfaip3$^{Lg\text{-}KO}$* mice treated with or without anti-IFNγ. '% cytokine of total:' indicates percentage all Th cells that produce one of the cytokines assayed. Symbols represent individual mice, A-B: n=6 mice per group, bars indicate mean values +/- SEM. Whitney U test two-tailed. AAI, allergic airway inflammation; HDM, house dust mite; WT, wild type; BAL: bronchoalveolar lavage. Source data are provided as a Source Data file

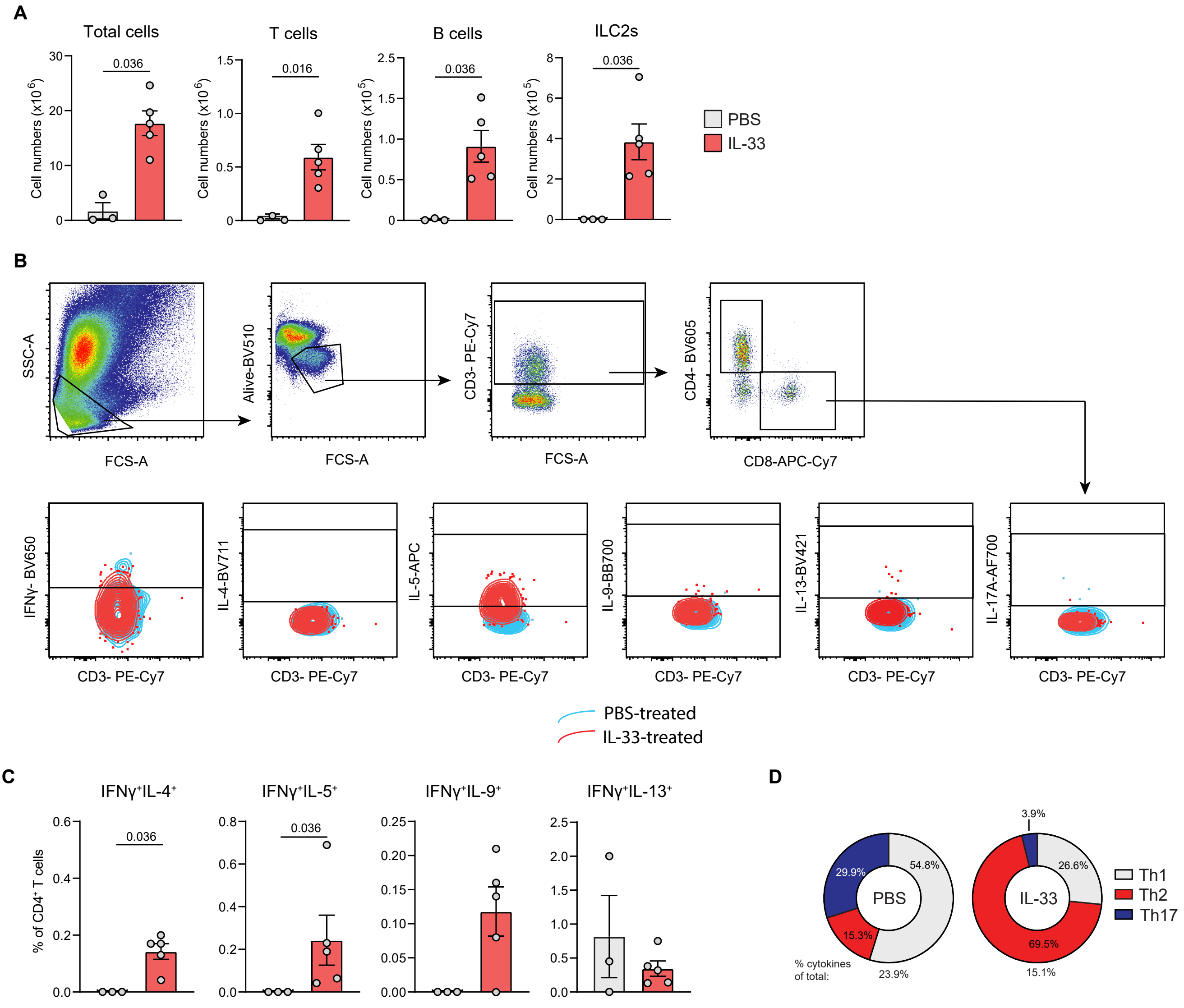

Figure S9. IL-33 induces type-2 skewing of Tc cells. (A) Numbers of total cells, T cells, B cells, and ILC2s determined in BAL by flow cytometry analysis. PBS: n=3 mice, IL-33: n=5 mice **(B)** Flow cytometry gating strategy used to identify cytokine production by Th and Tc cells in Figure 6D-G and S9C-D. **(C)** Quantification of IFNγ$^+$IL-4$^+$, IFNγ$^+$IL-5$^+$, IFNγ$^+$IL-9$^+$, and IFNγ$^+$IL-13$^+$ Th cells in BAL by flow cytometry. PBS: n=3 mice, IL-33: n=5 mice **(D)** Pie charts summarizing proportions of Th1, Th2 and Th17 cells in WT mice treated with PBS or IL-33. '% cytokine of total:' indicates percentage all Th cells that produce one of the cytokines assayed. Symbols represent individual mice, n=3-5 mice per group, bars indicate mean values +/- SEM. Mann-Whitney U test two-tailed. AAI, allergic airway inflammation; WT, wild type; BAL: bronchoalveolar lavage. Source data are provided as a Source Data file

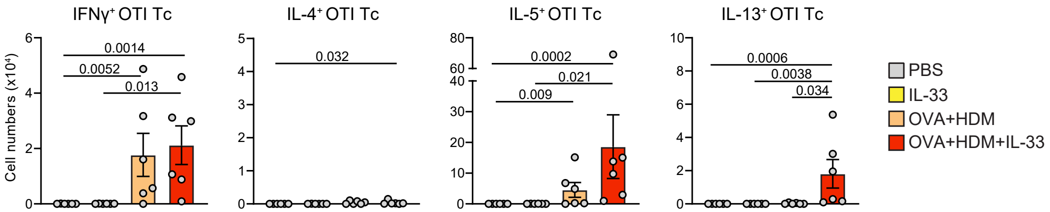

Figure S10. Type-2 skewing by IL-33 in the context of an antigen-specific Tc response. Numbers of IFNγ$^+$, IL-4$^+$, IL-5$^+$, and IL-13$^+$ OTI specific CD8 T cells in the BAL obtained by flow cytometry Symbols represent individual mice, n= 6 mice per group, bars indicate mean values +/- SEM. Kruskal-Wallis test corrected for multiple testing. BAL: bronchoalveolar lavage. Source data are provided as a Source Data file

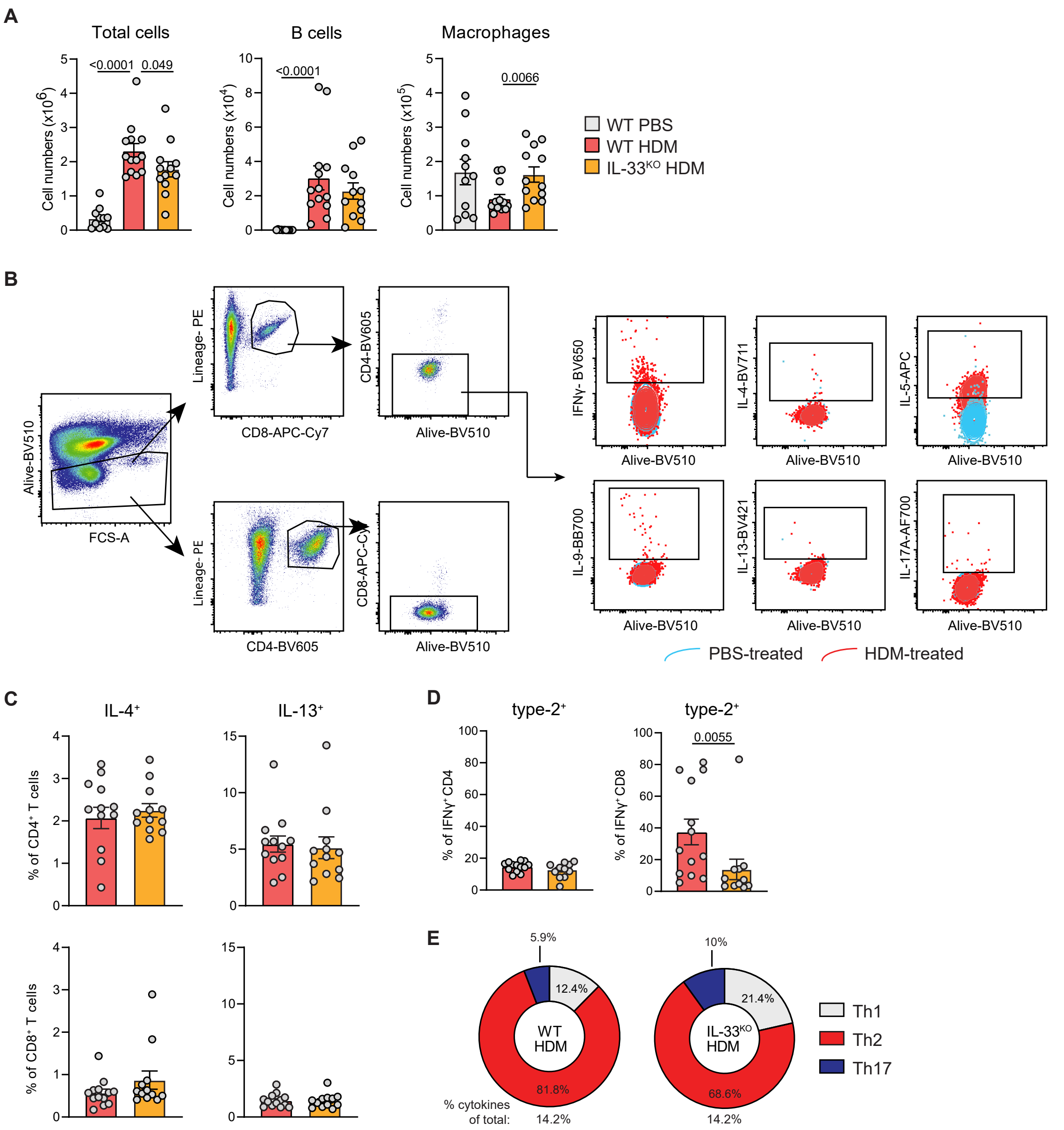

Figure S11. Loss of IL-33 reduces type-2 skewing in Tc cells. (A) Numbers of total cells, B cells, and macrophages determined in BAL by flow cytometry analysis. PBS: n=11, WT_HDM: n= 13, IL-33^{ko} HDM: n= 12 mice. **(B)** Flow cytometry gating strategy used to identify cytokine production by Th and Tc cells in Figure 8C-E and S11C-E. **(C)** Quantification of IL-4^{+} and IL-13^{+} Th and Tc cells in BAL by flow cytometry. WT_HDM: n= 13, IL-33^{ko} HDM: n= 12 mice. **(D)** Quantification of IFNγ^{+} Th and Tc cells also producing IL-5 or IL-9. WT_HDM: n= 13, IL-33^{ko} HDM: n= 12 mice. **(E)** Pie charts summarizing proportions of Th1, Th2 and Th17 cells in HDM-sensitized WT and IL-33^{KO} mice. '% cytokine of total:' indicates percentage all Th cells that produce one of the cytokines assayed. Symbols represent individual mice, bars indicate mean values +/- SEM. Mann-Whitney U test two-tailed. AAI, allergic airway inflammation; HDM, house dust mite; WT, wild type; BAL: bronchoalveolar lavage. Source data are provided as a Source Data file

Table S1. Asthma patient cohort characteristics.

	HC	Asthma (total)	Controlled (ACQ <0.75)	Partially controlled (ACQ 0.75-1.5)	Uncontrolled (ACQ >1.5)
	n=17	n=55	n=15	n=18	n=21
**Age (years)**	39.5 (±10.5)	45.3 (±14.2)	49.9 (±16.6)	38.6 (±12.7)	46.3 (±12.7)
**Sex (M/F)**	4/13	15/41	7/8	3/15	5/17
**BMI**	23.3 (±2.9)	28.2 (±5.5)	27.2 (±3.6)	28.0 (±6.2)	29.7 (±5.8)
**ACQ score**	NA**	1.4 (±0.9)***	0.45 (±0.24)	0.98 (±0.19)	2.4 (±0.58)
**%$FEV1_{post}$**	105.9 (±43.4)	89.8 (±13.9)	97.2 (±13.7)	90.1 (±13.5)	82.5 (±11.8)
**FeNO**	12.2 (±6.0)	23.3 (±21.5)	19.9 (±11.4)	19.1 (±9.9)	30.8 (±31.8)
**IgE (IU/ml)**	23.3 (±25.0)	289.8 (±520.6)	240.2 (±322.3)	200.5 (±308.4)	372.7 (±719.9)
**Exacerbations ******	NA	2 (±1.33)	1.3 (±1.53)	2.3 (±1.3)	2.35 (±1.09)
**Eosinophils (10^9/µl)**	0.09 (±0.068)	0.24 (±0.25)	0.23 (±0.17)	0.21 (±0.15)	0.21 (±0.22)
**ICS bioequivalents (*3*)**	NA	1362.2 (±975.9)	1000.5 (±722.2)	1470.5 (±936.3)	1820.9 (±1049.4)

Values represent means
± = Standard deviation
** Not Applicable
*** Ranging from 0 to 3.67
**** Registered number of exacerbations in year prior to inclusion

Table S2. Type-2 cytokine production by CD8 T cells between atopic and non-atopic, or between T2 and non-T2 asthma patients

	Atopic	Non-atopic	p-value	T2	Non-T2	p-value
**IL-4⁺ CD8**	2.66 (±0.25)	3.15 (±0.27)	0.19	15.63 (±4.30)	10.69 (±4.30)	0.48
**IL-5⁺ CD8**	2.32 (±0.38)	1.62 (±0.22)	0.11	0.71 (±0.08)	0.77 (±0.12)	0.71
**IL-9⁺ CD8**	1.65 (±0.20)	1.37 (±0.15)	0.27	0.71 (±0.06)	0.70 (±0.10)	0.93
**IL-13⁺ CD8**	1.63 (±0.18)	1.54 (±0.20)	0.74	8.74 (±0.97)	8.54 (±1.59)	0.92

Values represent means
± = Standard Error of Means

Table S3. Primers used in this study.

Target	Forward	Reverse
GATA3	CATTACCACCTATCCGCCCTATG	CACACACTCCCTGCCTTCTGT
IL5	ACATTGACCGCCAAAAAGAG	ATCCAGGAACTGCCTCGTC
IL4	CATCGGCATTTTGAACGAG	GACGTTTGGCACATCCATCT
IL13	ATTGCATGGCCTCTGTAACC	TGGGCTACTTCGATTTTGGT
IFNγ	GCAAAAGGATGGTGACATGA	TTCAAGACTTCAAAGAGTCTGAGG
T-bet	TCAACCAGCACCAGACAGAG	AAACATCCTGTAATGGCTTGTG
CRIPT	GTGGGAGGAAGCTGAACGAAA	CACATCGCACAGATGCCTTTT

Table S4. Anti-human antibodies used in this study.

Target	Conjugate	Clone	Manufacturer	Dilution
CD3	APC-Cy7	SK7	BD Biosciences	1:50
CD3	Biotin	UCHT1	Thermofisher	1:50
CD4	Biotin	OKT4	Thermofisher	1:50
CD4	FITC	RPA-T4	BD Biosciences	1:5
CD8	AF700	SK1	Biolegend	1:40
CD8	PerCP	SK1	Biolegend	1:20
CD14	Biotin	61D3	Thermofisher	2µl/100x10^6 cells
CD14	FITC	HCD14	Biolegend	1:50
CD16	Biotin	3G8	Thermofisher	6µl/100x10^6 cells
CD16	FITC	3G8	BD	1:50
CD19	Biotin	HIB19	Thermofisher	2µl/100x10^6 cells
CD19	FITC	HIB19	BD	1:50
CD36	Biotin	HM36	Biolegend	10µl/100x10^6 cells
CD45	AF700	30-F11	Biolegend	1:50
CD56	FITC	NCAM	Thermofisher	1:50
CD235ab	Biotin	QA20B11	Biolegend	2µl/100x10^6 cells
IFNγ	BV711	B27	BD Biosciences	1:20
IFNγ	BV786	4S.B3	BD Biosciences	1:50
IL-4	PE	8D4-8	Thermofisher	1:20
IL-5	APC	TRFK5	BD Biosciences	1:50
IL-9	BV421	MH9A3	BD Biosciences	1:10
IL-13	PE-Cy7	JES10-5A2	Biolegend	1:10
IL-13	PE	JES10-5A2	Biolegend	1:50
IL-17A	BV786	N49-653	BD Biosciences	1:20
CRTH2	PE-Cy7	BM16	Biolegend	1:20
LIVE/DEAD fixable Aqua	BV510	-	Thermofisher	1:2000

Table S5. Anti-mouse antibodies used in this study.

Target	Conjugate	Clone	Manufacturer	Dilution
CD3	PE-CF594	145-2C11	BD Biosciences	1:100
CD3	APC-eF780	17A2	Thermofisher	1:50
CD3	PE	145-2C11	Thermofisher	1:100
CD3	PE-Cy7	145-2C11	Biolegend	1:200
CD3	BV421	145-2C11	BD Biosciences	1:400
CD4	BV711	RM4-5	BD Biosciences	1:400
CD4	BV605	RM4-5	BD Biosciences	1:800
CD4	AF700	L3T4	Thermofisher	1:100
CD5	PE	53-7.3	Thermofisher	1:400
CD5	APC	Ly-1	Thermofisher	1:200
CD8	PE-Cy7	53-B6.7	Thermofisher	1:400
CD8	BV421	53-6.7	BD Biosciences	1:600
CD8	APC-EF780	53-6.7	Thermofisher	1:50
CD8	BV650	53-6.7	Biolegend	1:400
CD11b	PerCP-Cy5.5	M1/70	BD Biosciences	1:200
CD11b	Biotin	M1/70	Thermofisher	1:400
CD11b	PE	M1/70	Thermofisher	1:800
CD11b	APC	M1/70	Thermofisher	1:100
CD11c	PE-TxR	N418	Thermofisher	1:200
CD11c	Biotin	N418	Thermofisher	1:200
CD11c	PE	N418	Thermofisher	1:800
CD11c	BV786	HL3	BD Biosciences	1:100
CD11c	APC	N418	Thermofisher	1:100
CD19	APC-eF780	ID3	Thermofisher	1:50
CD19	Biotin	ID3	BD Biosciences	1:600
CD19	AF700	ID3	Thermofisher	1:200
CD44	PerCP-Cy5.5	IM7	Thermofisher	1:400
CD62L	APC	MEL-14	Thermofisher	1:100
CD103	eF450	2E7	Thermofisher	1:50
CD117	APC-Cy7	2B8	BD Biosciences	1:50
CD127	BV711	SB/199	BD Biosciences	1:100
B220	PE	RA3-6B2	Thermofisher	1:1600
B220	PE-Cy7	RA3-6B2	Thermofisher	1:1000
FceRIα	PE	MAR-1	Thermofisher	1:400
FceRIα	APC	MAR-1	Thermofisher	1:200
GATA3	APC	L50-823	BD Biosciences	1:50
GR-1	Biotin	1A8-Ly6g	BD Biosciences	1:800
GR-1	PE-Cy7	RB6-8C5	Thermofisher	1:800
GR-1	PE	RB6-8C5	BD Biosciences	1:1600
IL-4	BV711	11B11	BD Biosciences	1:100
IL-5	PE	TRFK-5	BD Biosciences	1:100
IL-5	Biotin	TRFK-5	BD Biosciences	1:200
IL-5	APC	TRFK-5	BD Biosciences	1:100
IL-9	PerCP-Cy5.5	D9302C12	BD Biosciences	1:50
IL-13	EF660	eBio13A	Thermofisher	1:200
IL-13	EF450	eBio13A	Thermofisher	1:100
IL-17A	AF700	TC11-18H10.1	BD Biosciences	1:100
IFNγ	EF450	XMG1.2	Thermofisher	1:400
IFNγ	BV650	XMG1.2	BD Biosciences	1:100
MHC class II	AF700	M5/114.15.2	Thermofisher	1:400
MHC class II	BV650	M5/114.15.2	BD Biosciences	1:2000
NK1.1	PE	NKR-P1C	Thermofisher	1:800
NK1.1	Biotin	PK136	Thermofisher	1:800
NK1.1	BV421	PK136	BD Biosciences	1:100
Sca-1	BV786	D7	BD Biosciences	1:800
Siglec-F	PE	E50-2440	BD Biosciences	1:1000
Siglec-F	APC-Cy7	E50-2440	BD Biosciences	1:50
Siglec-F	PE-TxR	E50-2440	BD Biosciences	1:400
ST2	FITC	DJ8	mdbioproducts	1:200
streptavidin	APC	-	Thermofisher	1:750
streptavidin	APC-Cy7	-	Thermofisher	1:200
T1ST2	Biotin	DJ8	MDBioproducts	1:200
T-bet	PE	Ebio4B10	Thermofisher	1:50
TCR Vα2	FITC	B20.1	Thermofisher	1:100
TCR Vα2	APC-Cy7	B20.1	BD Biosciences	1:100
TCR Vβ2	BV786	MR9-4	BD Biosciences	1:100
TER119	PE	TER119	Thermofisher	1:800
TER119	Biotin	Ly-76	BD Biosciences	1:1200
TER119	APC	TER119	Thermofisher	1:400
