# Supplementary material for: Type-2 CD8+ T-cell formation relies on interleukin-33 and is linked to asthma exacerbations
Source: Nat Commun. 2023 Aug 23;14:5137. doi: 10.1038/s41467-023-40820-x (PMC10447424; doi:10.1038/s41467-023-40820-x)
Supplement: Supplementary file 3 — Reporting Summary [file 41467_2023_40820_MOESM3_ESM.pdf]

nature portfolio

Corresponding author(s): Dr. Ralph Stadhouders, Dr. Rudi Hendriks

Last updated by author(s): Jun 30, 2023

Reporting Summary

Nature Portfolio wishes to improve the reproducibility of the work that we publish. This form provides structure for consistency and transparency in reporting. For further information on Nature Portfolio policies, see our Editorial Policies and the Editorial Policy Checklist.

Statistics

For all statistical analyses, confirm that the following items are present in the figure legend, table legend, main text, or Methods section.

n/a	Confirmed	
☐	☒	The exact sample size (*n*) for each experimental group/condition, given as a discrete number and unit of measurement
☐	☒	A statement on whether measurements were taken from distinct samples or whether the same sample was measured repeatedly
☐	☒	The statistical test(s) used AND whether they are one- or two-sided<br>*Only common tests should be described solely by name; describe more complex techniques in the Methods section.*
☐	☒	A description of all covariates tested
☐	☒	A description of any assumptions or corrections, such as tests of normality and adjustment for multiple comparisons
☐	☒	A full description of the statistical parameters including central tendency (e.g. means) or other basic estimates (e.g. regression coefficient) AND variation (e.g. standard deviation) or associated estimates of uncertainty (e.g. confidence intervals)
☐	☒	For null hypothesis testing, the test statistic (e.g. *F*, *t*, *r*) with confidence intervals, effect sizes, degrees of freedom and *P* value noted<br>*Give P values as exact values whenever suitable.*
☒	☐	For Bayesian analysis, information on the choice of priors and Markov chain Monte Carlo settings
☒	☐	For hierarchical and complex designs, identification of the appropriate level for tests and full reporting of outcomes
☐	☒	Estimates of effect sizes (e.g. Cohen's *d*, Pearson's *r*), indicating how they were calculated

Our web collection on statistics for biologists contains articles on many of the points above.

Software and code

Policy information about availability of computer code

Data collection	Flowcytometry data was collected using FACSDiva v9 software (BD Biosciences).
Data analysis	Flowcytometry data was analyzed using FlowJo v10 software (BD Biosciences). Statistical analyses were performed using GraphPad Prism software (v8).

For manuscripts utilizing custom algorithms or software that are central to the research but not yet described in published literature, software must be made available to editors and reviewers. We strongly encourage code deposition in a community repository (e.g. GitHub). See the Nature Portfolio guidelines for submitting code & software for further information.

Data

Policy information about availability of data

All manuscripts must include a data availability statement. This statement should provide the following information, where applicable:

- Accession codes, unique identifiers, or web links for publicly available datasets
- A description of any restrictions on data availability
- For clinical datasets or third party data, please ensure that the statement adheres to our policy

Source data depicted in all figures are provided with this paper (see Source data file.xlsx). Raw flowcytometry, quantitative PCR and ELISA data files generated and analyzed for the current study are available from the corresponding author upon reasonable request.

Field-specific reporting

Please select the one below that is the best fit for your research. If you are not sure, read the appropriate sections before making your selection.

☒ Life sciences ☐ Behavioural & social sciences ☐ Ecological, evolutionary & environmental sciences

For a reference copy of the document with all sections, see nature.com/documents/nr-reporting-summary-flat.pdf

Life sciences study design

All studies must disclose on these points even when the disclosure is negative.

Sample size	No sample size calculation was performed. Sample sizes were determined based on patient sample availability from previously conducted clinical studies or from experience with mouse models of (allergic) airway inflammation.
Data exclusions	No data was excluded in our analyses.
Replication	All attempts at replication were successful. All experiments were independently replicated at least 2 times.
Randomization	Samples or animals were randomized during the experiments.
Blinding	Investigators were not blinded during data collection or analysis.

Reporting for specific materials, systems and methods

We require information from authors about some types of materials, experimental systems and methods used in many studies. Here, indicate whether each material, system or method listed is relevant to your study. If you are not sure if a list item applies to your research, read the appropriate section before selecting a response.

Materials & experimental systems

n/a	Involved in the study	
☐	☒	Antibodies
☒	☐	Eukaryotic cell lines
☒	☐	Palaeontology and archaeology
☐	☒	Animals and other organisms
☐	☒	Human research participants
☒	☐	Clinical data
☒	☐	Dual use research of concern

Methods

n/a	Involved in the study	
☒	☐	ChIP-seq
☐	☒	Flow cytometry
☒	☐	MRI-based neuroimaging

Antibodies

Antibodies used	All antibodies, including Manufacturer, clone number and dilutions used are listed in Supplementary Tables 4 and 5.
Validation	All antibodies were validated by the manufacturers (see their websites for more details). Additional validation was performed in-house through titration experiments involving known negative and positive cell populations (e.g. CD3 tested on splenic cells which contain T cells [positive] and B cells [negative]).

Animals and other organisms

Policy information about studies involving animals; ARRIVE guidelines recommended for reporting animal research

Laboratory animals	All mice were housed and bred under SPF conditions at the Erasmus MC and analyzed at 6-12 weeks of age after being euthanized by an overdose of pentobarbital. Housing temperature was between 19-24 degrees Celsius, humidity was between 40-70% and mice were placed on a fixed light (7AM-19PM) - dark cycle. In all experiments, we strived for a 1:1 male/female ratio; non-transgenic littermates were used as WT controls. Experimental and control animals were co-housed. WT and OTI/OTII (carrying an OVA-specific transgenic TCR) mice were purchased from Envigo (WT strain: #680) and Jackson (OTI strain: #003831, OTII strain: #004194) respectively. Tnfaip3fl/fl mice were crossed to Cd207CRE/+ (Langerin-CRE) mice to generate Tnfaip3Lg-KO mice 37. The Gata3YFP/YFP (GATIR) mouse strain 62 was crossed to the Foxp3-IRES-mRFP (FIR) reporter mice strain 63 to obtain Gata3YFP/YFPFoxp3IRES/mRFP mice. IL33KO mice 64 were crossed with Gata3YFP/YFPFoxp3IRES/mRFP mice. All mice were backcrossed to the C57BL/6 genetic background for at least six generations. Please contact the corresponding author if interested in obtaining these mouse strains. All experiments were performed with approval by the animal ethics committee of the Erasmus MC under a permit licensed by the Dutch government (AVD101002016637).
Wild animals	No wild animals were used in this study.

Field-collected samples	No field-collected samples were used in this study.
Ethics oversight	All experiments were performed with approval by the animal ethics committee of the Erasmus MC under a permit licensed by the Dutch government.

Note that full information on the approval of the study protocol must also be provided in the manuscript.

Human research participants

Policy information about studies involving human research participants

Population characteristics	see Supplementary Table 1. Smokers (>10 pack years), obese patients (BMI>35) and patients whom received systemic corticosteroid therapy three months prior to inclusion were excluded. Healthy controls were age- and sex-matched.
Recruitment	Asthma patients and healthy controls were recruited by the Franciscus Gasthuis & Vlietland hospital in Rotterdam as previously described (ref.7). Sex/gender (self-reported) was not considered for recruitment of patients, although for healthy control inclusion we aimed for individuals that were age- and sex-matched. We are not aware of any potential (self-selection) biases.
Ethics oversight	All experimental procedures were reviewed and approved by the Medical Ethical Committees of the Franciscus Gasthuis & Vlietland and the Erasmus MC.

Note that full information on the approval of the study protocol must also be provided in the manuscript.

Flow Cytometry

Plots

Confirm that:

☒ The axis labels state the marker and fluorochrome used (e.g. CD4-FITC).

☒ The axis scales are clearly visible. Include numbers along axes only for bottom left plot of group (a 'group' is an analysis of identical markers).

☒ All plots are contour plots with outliers or pseudocolor plots.

☒ A numerical value for number of cells or percentage (with statistics) is provided.

Methodology

Sample preparation	For both human and mouse: dead cells were excluded using Fixable viability dye (eBioscience). Cells were stained extracellular for 30 minutes at 4°C. For intracellular cytokine stainings, cells were stimulated with PMA (10 ng/mL; Sigma) and Ionomycin (500 nM; Merck) in the presence of GolgiStop (BD) for 4 hours at 37°C. Cells were fixed with PFA (2%, 10 minutes, 4°C) and permeabilized with Saponin (0.5%, 30 minutes at RT). For mouse intracellular transcription factor measurements, cells were fixed and permeabilized using the eBioscience Foxp3/transcription factor staining buffer set (Thermo Fisher Scientific) according to the manufacturer's instructions.
Instrument	Data were acquired on LSR II or FACSymphony flow cytometers (BD Biosciences).
Software	Flowcytometry data was collected using FACSDiva v9 software (BD Biosciences). Flowcytometry data was analyzed using FlowJo v10 software (BD Biosciences).
Cell population abundance	Cell purities were validated using flowcytometry measurements and were >99%.
Gating strategy	also see Supplementary Figure 1A/6E/7B/9B/11B for representative gating strategies.<br><br>For the quantification of cytokine production by human CD4 and CD8 T cells we used the following gating strategy: We pre-gate on the time-gate, including only a stable FCS-A signal throughout the measurement. Then lymphocytes are gated based on the SSC-A/FSC-A profile. From this, dead cells (LIVE/DEAD fixable Aqua positive cell) and doublets (FSC-W/FSC-A) are excluded. Th cells are gated as CD3+CD4+CD8- cells and Tc cells are gated as CD3+CD8+CD4-. From this cytokine expression is gated. The separation between the positive and the negative cloud is clearly visible and positive/negative boundaries are defined based on this separation. (Figure S1A)<br>For the quantification of cytokine production by murine CD4 and CD8 T cells we used the following gating strategy: We pre-gate on the time-gate, including only a stable FCS-A signal throughout the measurement. Then lymphocytes are gated based on the SSC-A/FSC-A profile. From this, dead cells (LIVE/DEAD fixable Aqua positive cell) and doublets (FSC-W/FSC-A) are excluded. Th cells are gated as CD3+CD4+CD8- cells and Tc cells are gated as CD3+CD8+CD4-. From this cytokine expression is gated. The separation between the positive and the negative cloud is clearly visible and positive/negative boundaries are defined based on this separation. As an extra control, T cells from PBS treated mice rarely make cytokines, these populations are used as negative boundaries (Figure S6E, S7B, S9B).<br><br>For the IL-33KO-HDM experiment we used the following gating strategy: We pre-gate on the time-gate, including only a stable FCS-A signal throughout the measurement. Then lymphocytes are gated based on the SSC-A/FSC-A profile. From this, dead cells (LIVE/DEAD fixable Aqua positive cell) and doublets (FSC-W/FSC-A) are excluded. Th cells are gated as lineage+CD4+CD8- cells and Tc cells are gated as lineage+CD8+CD4-. From this cytokine expression is gated. The separation between the

positive and the negative cloud is clearly visible and positive/negative boundaries are defined based on this separation. As an extra control, T cells from PBS treated mice rarely make cytokines, these populations are used as negative boundaries. (Figure S11B)
For the cell populations present in the bronchial lavages we pre-gated on the time-gate, including only a stable FCS-A signal throughout the measurement. Then debris is gated out based on the SSC-A/FSC-A profile. From this, dead cells (LIVE/DEAD fixable Aqua positive cell) and doublets (FSC-W/FSC-A) are excluded.

Macrophages are gated as FITC+ auto-fluorescent cells.
Eosinophils are gated as FITC-SiglecF+
Neutrophils are gated as FITC-GR1hi
Dendritic cells are gated as FITC-SiglecF-GR1-CD11c+MHCII+
cDC1 cells are gated as FITC-SiglecF-GR1-CD11c+MHCII+CD103+CD11b -
cDC2 cells are gated as FITC-SiglecF-GR1-CD11c+MHCII+CD11b+CD64-
moDC cells are gated as FITC-SiglecF-GR1-CD11c+MHCII+CD11b+CD64+
B cells are gated as FITC-SiglecF-GR1-CD19+MHCII+
T cells are gated as FITC-SiglecF-GR1-CD3+MHCII-

☒ Tick this box to confirm that a figure exemplifying the gating strategy is provided in the Supplementary Information.
